# The Relations of Preventive Medicine to Political Economy

**Published:** 1899-04

**Authors:** 


### The Relations of Preventive Medicine to Political Economy.

Dr. George W. Brush, of Brooklyn, discussed this subject. He pointed out that in New York State alone there were at least thirteen thousand deaths from pulmonary tuberculosis, and that the average number of deaths annually in New York City alone from this same disease was 6,072. As yet only one State, Massachusetts, had seen fit to provide a hospital for its consumptives. It was certainly the duty of the State Society to urge the Legislature to adopt a similar course, and also to make a strong effort to diffuse among the laity a better knowledge of the nature of this terrible scourge, and the best methods of checking its ravages.—*Ex.*

